# Un accident vasculaire hémorragique révélant une méningite à *Neisseria meningitides*

**DOI:** 10.11604/pamj.2015.20.134.5994

**Published:** 2015-02-16

**Authors:** Mamoudou Savadogo, Georges Rosario Christian Millogo, Sylvain Zabsonré

**Affiliations:** 1 Service des Maladies infectieuses du Centre Hospitalier Universitaire Yalgado Ouédraogo, Ouagadougou, Burkina Faso; 2 Service de Cardiologie du Centre Hospitalier Universitaire Yalgado Ouédraogo, Ouagadougou, Burkina Faso; 3 Service de Neurochirurgie du Centre Hospitalier Universitaire Yalgado Ouédraogo, Ouagadougou, Burkina Faso

**Keywords:** Accident vasculaire hémorragique, méningite, Neisseria meningitidis, stroke, meningite, Neisseria meningitidis

## Abstract

L'accident vasculaire hémorragique et la méningite purulente constituent des urgences médicales chez la personne âgée. L'Objectif est de rapporter un cas d'accident vasculaire hémorragique associé à une méningite purulente à Neisseria meningitidis survenus chez une personne âgée. Il s'agit d'une patiente de 62 ans sans antécédent pathologique connu, a été admise aux urgences médicales du CHU YO pour céphalées + vomissement et notion de perte brutale de connaissance. L'examen à son admission notait un syndrome infectieux, une conscience obnubilée sans déficit moteur, une raideur cervicale et une hypertension artérielle. La tomodensitométrie cérébrale avait objectivé une hémorragie méningée diffuse avec inondation du 4^ème^ ventricule responsable d'une hydrocéphalie triventriculairesous jacente d'allure obstructive associée à des sinusites ethmoïdo-sphénoïdales droites chroniques. Une ponction lombaire réalisée avait montré un liquide cérébrospinal citrin dont l'analyse était en faveur d'une méningite purulente à méningocoque. La glycémie était élevée à 15,8 mmol/l et l'hémoglobine glyquée était égale à 7,7%. L’évolution a été favorable sous traitement à base de ceftriaxone et de Metformine. Devant un cas suspect de méningite chez une personne âgée, la ponction lombaire après un TDM cérébral permet de poser le diagnostic et d'entreprendre rapidement une antibiothérapie indispensable pour la prévention des complications.

## Introduction

L'accident vasculaire hémorragique est une pathologie relativement fréquente en Afrique [1, 2]. Parmi les facteurs favorisant, il y a l'hypertension artérielle et le diabète [2–4]. Mais ilpeut survenir au décours d'une infection comme la méningite cérébro-spinale [2]. L'hémorragie méningée se caractérise alors par une issue de sang dans l´espace sous-arachnoïdienconsécutive à la rupture d'un vaisseau sanguin. Elle peut aussi être due à une pression artérielle élevée non maîtrisée. C'est une urgence médicale voire médico-chirurgicaleet l'hospitalisation rapide dans une structure adaptée constitue un facteur essentiel du pronostic. Nous rapportons un cas de complication d'une méningite à méningocoque dont l'objectif est de décrire les caractéristiques cliniques épidémiologiques tomodensitométriques thérapeutiques et évolutives.

## Patient et observation

**Figure 1:**
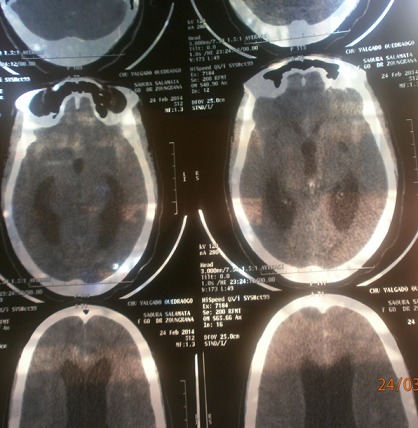
Scanner cérébral

Une patiente âgée de 62 ans sans antécédent pathologique connu, a été admise aux urgences médicales du Centre hospitalier universitaire Yalgado Ouédraogo le 28/02/2014 pour céphalées+vomissement et notion de perte brutale de connaissance. L'examen clinique à l'admission notait une conscience obnubilée avec un Glasgow à 14, une TA=130/90mmHg, une température=38°7, la fréquence cardiaque=96battements/mn. L'examen du système nerveux ne notait pas de déficit moteur mais il y avait une raideur cervicale et une désorientation temporo spatiale. La tomodensitométrie cérébrale demandé a objectivé une hémorragie méningée diffuse avec inondation du 4^ème^ ventricule responsable d'une hydrocéphalie triventriculaire sous-jacente d'allure obstructive; des sinusites ethmoïdo-sphénoïdales droites chroniques; une lacune capsulo-lenticulaire gauche probablement d'accident vasculaire ancient et un œdème cérébral diffus (Figure 1). Une ponction lombaire réalisée avait montré un liquide céphalorachidien citrin dont l'examen cytobactériologique notait des leucocytes supérieur à 1000/mm3 (polynucléaire neutrophile 58%, lymphocyte 42%; des hématies 200/mm3) et le test au latex était positif au méningocoquede sérogroupe W.

La glycémie était élevée à 15,8 mmol/l et l'hémoglobine glyquée était égale à 7,7%. Le reste de l'examen biologique était sans particularité. Sous traitement à base de ceftriaxone2g/J, de Metformine 500mg 1cpx2/j, l’évolution a été favorable et elle est sortie de l'hôpital le 17 Mars 2014.

## Discussion

Les accidents vasculaires hémorragiques représentent 20% de l'ensemble des accidents vasculaires. Ils sont provoqués par la rupture d´une malformation vasculaire dans 60 à 90% [5]. Leur pronostic est variable selon l´étiologie. Ils peuvent être extrêmement graves et mettre en jeux le pronostic vital, d´où la nécessité d'un bilan et d´un traitement en milieu spécialisé [5]. Leurs etiologies sont dominées par l'anévrisme [6] intracrânien, mais ils peuvent se rencontrer au cours de nombreuses affections comme une méningite purulente comme relevé par Barquet [7]. Contrairement aux autres types d'accidents vasculaires cérébraux, il existe une prédominance féminine de l'hémorragie méningée dont le risque s'augmente avec la présence de certains facteurs de risque cardiovasculaire comme l’âge le diabète et l'hypertension artérielle dont souffrait notre patiente [7]. La Tomodensitométrie cérébrale avait objectivé outre l'hémorragie méningée, des sinusites qui sont souvent les portes d'entrée des méningites purulentes comme déjà relevé parGehanno à Paris et Eholié à Abidjan [8–10]. Notre patiente souffrait d'un diabète qui était méconnu de la patiente. Elle était également hypertendue non suivi et la tomodensitometrie cérébrale (TDM) avait objectivé une lacune capsulo-lenticulaire faisant suspecter un accident vasculaire ancien. Les accidents vasculaires cérébraux sont rarement hémorragiques chez le diabétique en dépit de l'augmentation de la fréquence de l'hypertension artérielle. Par contre, les micro-infarctus responsable de lacune semblent plus fréquents chez le diabétique en particulier en cas d'association diabète et hypertension artérielle.

## Conclusion

Les méningites purulentes sont des pathologies pour lesquelles la précocité du diagnostic et du traitement conditionne indiscutablement le pronostic vital et fonctionnel. Les patients ayant des facteurs de risque cardiovasculaire comme l’âge, le diabète et l'hypertension artérielle, sont exposées à des accidents vasculaires hémorragiques. Devant un cas suspect de méningite chez une personne âgée, la ponction lombaire après un TDM cérébral permet de poser le diagnostic et d'entreprendre rapidement une antibiothérapie indispensable pour la prevention des complications hémorragiques. Le développement d'un vaccin quadrivalent (ACYW) conjugué contribuera à réduire considérablement la prévalence des méningites et leurs complications hémorragiques qui sont graves.
